# The consequences of fermentation metabolism on the qualitative qualities and biological activity of fermented fruit and vegetable juices

**DOI:** 10.1016/j.fochx.2024.101209

**Published:** 2024-02-10

**Authors:** Shah Saud, Tang Xiaojuan, Shah Fahad

**Affiliations:** a College of Life Science, Linyi University, Linyi, Shandong 276000, China; b Department of Agronomy, Abdul Wali Khan University Mardan, Mardan, Pakistan; c Department of Natural Sciences, Lebanese American University, Byblos, Lebanon

**Keywords:** Fruits and vegetables, Fermentation, Microbial growth, Active substance, Probiotics

## Abstract

Fermentation of fruit and vegetable juices with probiotics is a novel nutritional approach with potential health benefits. Lactic acid fermentation-based biotransformation results in changes in the profile and nature of bioactive compounds and improves the organoleptic properties, shelf life and bioavailability of vitamins and minerals in the fermented juices. This process has been shown to enrich the phenolic profile and bioactivity components of the juices, resulting in a new type of functional food with improved health benefits. Fruits and vegetables are the ideal substrate for microbial growth, and fruit and vegetable juice will produce rich nutrients and a variety of functional activities after fermentation, so that the high-quality utilization of fruits and vegetables is realized, and the future fermented fruit and vegetable juice products have a wide application market. This paper explores the typical fermentation methods for fruit and vegetable juices, investigates the bioactive components, functional activities, and the influence of fermentation on enhancing the quality of fruit and vegetable juices. The insights derived from this study carry significant implications for guiding the development of fermented fruit and vegetable juice industry.

## Highlights

• Influence of fermentation on quality improvement of fermented fruit and vegetable juices.
• Function of Lactobacillus and the selection of Lactobacillus for fermented fruits and vegetables.
• Improve the flavor characteristics of fruit and vegetable juice products.

## Graphical abstract

**Figure:**
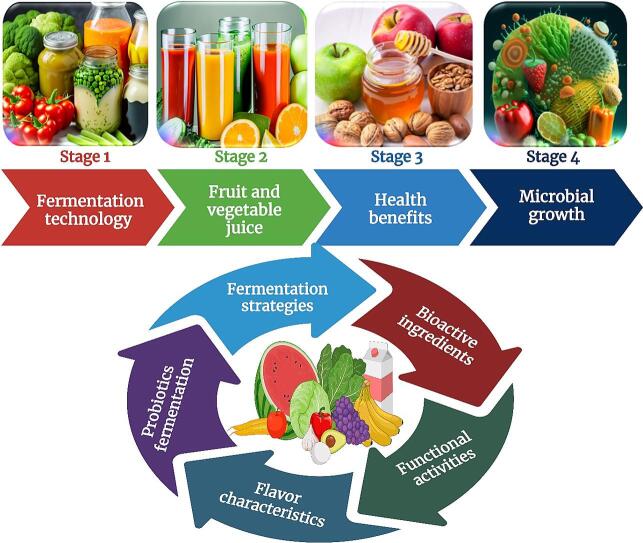

## Introduction

Fruit and vegetable resources are rich, but there are some disadvantages such as low storage and processing capacity and strict fresh sales. This leads to the waste of raw materials for fruits and vegetables, so further deep processing is required to solve the above problems. The use of probiotic fermentation technology to develop fermented fruit and vegetable juice drinks with special nutritional and health functions can not only eliminate the waste of resources, improve the taste of fruit and vegetable products, however also improve their health function and enrich the color variety of fruit and vegetable products, solving the bottleneck problem that restricts the development of the fruit and vegetable processing industry (Žuntar et al., 2020, Guan et al., 2020). As a kind of nutritious food with more development potential, lactic acid bacteria-fermented fruit and vegetable juices can produce a variety of amino acids, vitamins, digestive enzymes and other substances necessary for the human body through the fermentation of lactic acid bacteria and yeast. It not only provides a pleasant flavor and fragrance, but also gives fruit and vegetable juices an elegant and balanced lactic acid fermentation taste, promotes the absorption of iron and vitamin D, and improves food digestibility and the utilization rate of calcium, phosphorus and iron (Hirose et al., 2006 Dec, Devine and Hancock, 2002). Studies have shown that fruit and vegetable juices fermented with lactic acid bacteria have nutritional and health functions such as: regulating the gastrointestinal flora of the body maintaining of microecological balance, regulation of immunity, antioxidants, inhibition of tumor cell growth and lowering of cholesterol activity and biological antagonism in vitro (Elmadfa et al., 2010). Compound fermented fruit and vegetable juices combine a variety of effects and have broad market prospects. Fermentation strains, fermentation raw materials and new functions of fermented fruit and vegetable juices will be important topics in the development and research of fruit and vegetable products in the future (Sun et al., 2022, Garcia et al., 2020).

Fruits and vegetables are rich in carbohydrates, vitamins, minerals, phenolic compounds and other nutrients are internationally recognized as healthy foods. Many studies have proved that eating fruits and vegetables can reduce the risk of chronic diseases (Urbonaviciene et al., 2015, Di Cagno et al., 2013), which is why more and more consumers prefer natural fruit and vegetable products. However, due to the relatively high-water content of fresh fruits and vegetables, it is easy to rot due to the proliferation of microorganisms, which poses a great challenge for transportation and storage. In recent years, fruits and vegetable production has increased with the development of planting technology and optimization of varieties, however also made a large number of high-quality fruits and vegetables varieties unsaleable, resulting in serious waste of resources (Swain et al., 2014). On the other hand, due to backward processing technology, the current fruit and vegetable products are mainly fresh sales and primary processed products (such as cans, fruit and vegetable juices), which is the nature of the development of retail, and deep processing fruit and vegetable industries is extremely slow, it’s becoming more and more difficult to meet consumer needs. Fermentation is a simple, low-cost, and sustainable technology that extends the shelf life of fruits and vegetables (Paramithiotis et al., 2022, Urbonaviciene et al., 2015). The fermentation of fresh fruit and vegetable juice not only retain essential nutrients and degrade toxic compounds, however also creates number of new active ingredients that are beneficial to health, improve the taste of food and thus increase the added value and utilization of fruits and vegetables. The survey shows that probiotic products occupy 65 % of the world functional food market share and that fermented fruit and vegetable juice has emerged as a new popular probiotic product due to its significant health benefits and good flavor (Küçükgöz et al., 2022). In recent years, with the deepening research on the technology of probiotic fermented fruit and vegetable products, reports on the use of probiotics in the processing of fruit and vegetable beverages are also increasing (Mojikon et al., 2022, Žuntar et al., 2020). The use of probiotic biological fermentation technology in the development of nutritious and functional fermented fruit and vegetable juice products is an extension of deep processing technology for fruits and vegetables. This technology not only extends the shelf life of fruit and vegetable juices but also inhibits the growth of hybrid bacteria, resulting in better fermentation flavor, nutritional value, and greater product variety (Kiczorowski et al., 2022). It has solved the bottleneck problem that restricts the development of fruit and vegetable processing industry. Japan, the United States, France, Germany, South Korea and other countries have taken a leading position in the research and development of fermented fruit and vegetable juice, especially Japan, in the research and application of probiotic fermented fruit and vegetable juice. Probiotic fruit and vegetable products at home and abroad are developing diversified and the range of products line is constantly expanding (Bagchi, 2014). Based on the above development status, In this paper, the selection of fermented juice strains, fermentation technology and nutritional value of research progress, and discuss its development prospects, in order to provide reference for the research and development of fermentation on nutritional and functional properties of fruit and vegetable juices.

## Fermentation techniques for elevating the quality of fruit and vegetable juices

Fermentation is a key biological transformation process in which microorganisms are used to obtain valuable metabolites. This complicated process has the potential to improve the safety, nutritional content, sensory properties and shelf life of fruits and vegetables, positioning it as a straightforward and environmentally friendly biotechnological practice (Di et al., 2013). The versatility of fermentation is demonstrated by various methods, including natural fermentation, inoculation with endogenous strains, and fermentation with commercially available strains, each providing different benefits (Skowron et al.,2022).

**Fig. 1:**
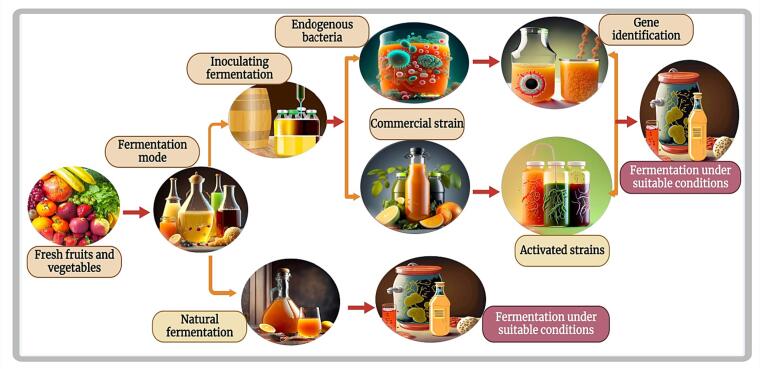
Techniques utilised for the fermentation strain of fruit and vegetable juice.

As depicted in (Fig. 1), the methods used in fermentation play a crucial role in the outcome of the process. In natural fermentation, the microorganisms inherent in the raw materials are used, while in inoculated endogenous strains, specific microorganisms are specifically introduced (Sharma et al., 2020). Commercial strains, on the other hand, use strains that are specifically designed for optimal fermentation results. In (Table 1) a comprehensive presentation of these three different fermentation approaches is provided and highlights the special product properties that result from the fermentation of various fruit and vegetable raw materials. The table outlines how each method impacts the final product and provides insight into the nuanced effects on flavor profiles, nutritional improvements, and overall quality. By examining the peculiarities of fermentation methods, a deeper understanding of the dynamic interaction between microorganisms and raw materials is created and underlines the importance of fermentation as a transformative and sustainable biotechnological process (Sun et al.,2022).

**Table 1:** Methods of Fermentation fermented fruit and vegetable juices.

Fermentation method	Ingredients	Fermentation strain	Main feature	References
Complex strain	Apricot Apple Pomegranate Pear Bergamot	*Lactobacillus and Bifidobacterium* *Saccharomyces cerevisiae and Lactobacillus* *Plantarum* *Lactobacillus plantarum and Lactobacillus acidophilus* *Lactobacillus plantarum, Lactobacillus Swiss and Lactobacillus casei* *Three kinds of Lactobacillus plantarum*	The mixed strain fermentation samples showed higher numbers of viable bacteria after fermentation Fermentation process introduced new volatile components that greatly enhanced the taste of the apple juice. The soluble solids in the apple juice decreased to 4.63 %, while the titratable acidity increased to 0.92 mg/mL The mixed fermentation of *Lactobacillus plantarum* and *Lactobacillus casei* had strong 2,2-Diphenyl-1-picrylhydrazyl free radical scavenging ability pH, glucose and fructose consumption, lactic acid production and 2,2-Diphenyl-1-picrylhydrazyl scavenging effect of binary fermentation were significantly improved	Bujna et al., 2017, Chen et al., 2023, Chen et al., 2023, Mantzourani et al., 2022, Sharma et al., 2022, Hashemi, et al., 2017
Endogenous strain fermentation	Blueberry Tomato Carrots, beans and zucchini	*Plant lactic acid bacteria, Lactobacillus fermentans* *Lactobacillus plantarum* *Lactobacillus plantarum, Lactobacillus brevis, micrococcus pentosus, etc*	Lactic acid was markedly elevated when the number of viable bacteria exceeded 10.0 log CFU/mL. Total phenolic content increased by 7.1–85.5 %, in vitro antioxidant capacity increased by 38.0 % The number of viable bacteria was significantly higher than that of exogenous strains, and the content of ascorbic acid, glutathione and total antioxidant activity of tomato juice fermented by endogenous *Lactobacillus plantarum were* also higher during storage pH drops rapidly and carbohydrate consumption is high	Barbosa et al., 2022 Di Cagno et al., 2009 Jan 15, Cui et al., 2019
Single strain	Apple Sweet potato Grapefruit	*Lactobacillus plantarum, Lactobacillus Swiss, etc* *Bifidobacterium, etc* *Lactobacillus plantarum, Lactobacillus fermentans*	*Lactobacillus plantarum* and *Lactobacillus acidophilus* showed a strong ability to convert malic acid into lactic acid Soluble dietary fiber, total polyphenol, organic acid content and stability of sweet potato residues were improved after fermentation pH decreased significantly, acidity and V_C_ increased significantly	Wang et al., 2021, Zhu et al., 2022, Luan et al., 2021
Natural fermentation	Rosa roxburghii Red raspberry persimmon	*Saccharomyces cerevisiae, Micrococcus lactis,* *Sphingosinomonas acanthoderma* Pichia and Saccharomyces lactic acid bacteria, acetic acid bacteria, yeast, etc	The total acid content of fruit wine was increased, the reducing sugar content was decreased, and the antioxidant capacity was significantly improved. Promote the production of enzymes and polysaccharides and other active substances Superoxide anion scavenging activity was significantly increased by 20.15 %, and total flavonoids and polyphenols were also significantly increased	Wispen et al., 2022, Yao et al., 2020, Ruan et al., 2022

### Harnessing nature's fermentation for fresh fruit and vegetable

Natural fermentation is a process in which native bacteria in raw materials ferment without artificial inoculation of bacteria. This process leads to the growth of various lactic acid bacteria, which leads to variations in taste and quality of the fermented products. Biotransformation based on lactic acid fermentation changes the profile and nature of bioactive compounds and improves the organoleptic properties, shelf life and bioavailability of vitamins and minerals in the fermented juices. Natural fermentation of fresh fruits and vegetables can occur under suitable conditions of moisture and temperature (Shen et al., 2023) used prickly pear as a raw material to produce prickly pear fruit wine through natural fermentation. The results showed that fermentation significantly increased the total acid content of fruit wine, reduced reducing sugar content, and significantly increased antioxidant capacity. After the natural fermentation of red raspberries, active ingredients such as enzymes and polysaccharides were produced and the microbial composition of red raspberries was analyzed through high-throughput sequencing, laying the foundation for its industrial production (Ruan et al., 2022) studied the changes of microbial composition and flavor substances during the natural fermentation of persimmon vinegar (Song et al., 2019) results showed that the main bacteria at the subordinate level were *Burkholderia*, *Lactobacillus* and *Acetobacter*. The most important true bacteria are *Saccharomyces* and *Cladosporium*. After fermentation, the superoxide anion radical scavenging activity was significantly increased by 20.15 % and the contents of total flavonoids and polyphenols was also significantly increased. Although natural fermentation as a traditional fermentation technology has brought great economic benefits, there are still many factors limiting its development, such as long fermentation time, poor inhibition of spoilage and pathogenic bacteria, unknown fermentation flora leading to product contamination, short shelf life etc. Unpredictability of sensory quality of fermented products. Therefore, it makes more sense to control the fermentation process by inoculation fermentation.

### Exploring endogenous strain Fermentation: Detection and technological characterization“

The final quality and flavor of fermented fruit and vegetable juice mainly depends on the selection of starter culture. Each fruit and vegetable has its unique dominant flora in the fermentation process, which is influenced by the variation in its own physical and nutritional conditions. Studies have shown the using unique flora isolated from raw materials as a starter favors fermentation. (Hosseini et al., 2018; Li et al., 2021) isolated plant lactic acid bacteria and fermenting *Lactobacillus* from tomatoes, blueberries and other fruits and inoculated them into blueberry juice for fermentation. After 48 h of fermentation, the viable bacteria count exceeded 10.0 log CFU/mL, the lactic acid content increased to 2190.91 mg/L, and the total phenolic content increased by 6.6 %-86.4 %. In vitro antioxidant capacity increased by 36.0 %. (Di et al., 2009) compared the starter culture of *Lactobacillus plantarum* isolated from tomatoes with the exogenous strain. The results indicate that the exogenous strain exhibited a prolonged incubation period for both growth and acidification. Following a 17-hour fermentation period at 25 °C, the viable bacteria count of the endogenous strain increased from approximately 7.6 log CFU/mL to 9.7 log CFU/mL. The content of *Lactobacillus plantarum* was about 8.8 log CFU/mL. In addition, endogenous fermented tomato juice, from *Lactobacillus plantarum* also had higher ascorbic acid, glutathione content and antioxidant activity during storage compared to exogenous fermented tomato juice from *Lactobacillus plantarum*. The results showed that the pH of the endogenous strain decreased faster after fermentation and carbohydrate consumption was higher than that of the exogenous strain. There were also significant differences between endogenous and exogenous strains in vitamin C concentration, color index, hardness and sensory properties (Cui et al., 2019). The endogenous strains have strong adaptability to fermentation environment and the fermented fruit and vegetable juice has high nutritional value, but the process of selecting suitable endogenous strains for specific fruit and vegetable fermentation is complicated and the cycle is long.

### Optimizing fruit and vegetable juice fermentation with commercial strains“

**Fig. 2:**
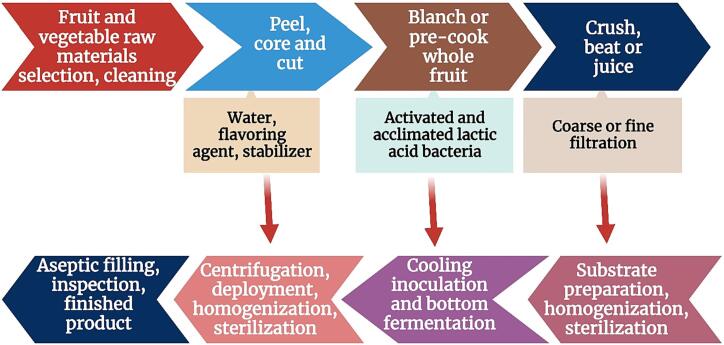
Process flow chart of lactic acid bacteria fermentation of fruit and vegetable juice.

Commercial starter cultures are standardized inoculants used in the manufacture of fermented foods under strict quality assurance and quality control. By using commercial strains, the entire fermentation process can be better controlled and the risk of fermentation errors can be minimized (Swain et al., 2014; Tang et al., 2022). Currently, the main commercial strains used in fruit and vegetable fermentation are lactic acid bacteria such as *Lactobacillus plantarum*, *Lactobacillus casei*, *Lactobacillus acidophilus* and *Lactobacillus Swiss*, followed by yeast and acetic acid bacteria. These commercial starter cultures typically use single or mixed strain fermentation to produce fruit and vegetable fermentation products. Compared with natural fermentation, the use of single-strain fermentation can greatly shorten the fermentation time and is more conducive to industrial production. (Wang et al., 2021) selected six kinds of lactic acid bacteria, including *Lactobacillus plantarum* and *Lactobacillus Swiss*, to ferment Fuji apple juice. The results showed that Fuji apple juice was a good fermentation substrate for lactic acid bacteria. The production process of fruit and vegetable juice fermented with lactic acid bacteria is shown in (Fig. 2). The maximum number of viable bacteria reached 12.7 log CFU/mL after fermentation, and *Lactobacillus plantarum* and *Lactobacillus acidophilus* showed a strong ability to convert malic acid to lactic acid. The resulting lactic acid is more than 6.5 mg/mL. (Zhu et al., 2022) used four lactic acid bacteria (Table 2) and one bifidobacterium to independently ferment sweet potato residues, and evaluated its nutritional composition, antioxidant activity, volatile substances and stability. The results showed that the soluble dietary fiber, total phenol, organic acid and the stability of sweet potato residues were improved after fermentation. (Quan et al., 2022) selected *Lactobacillus plantarum* and *Lactobacillus fermentum* to ferment pomelo juice respectively. The results showed that pomelo juice was an excellent substrate for the growth of two kinds of lactic acid bacteria; the number of viable bacteria each exceeded 8 logCFU/mL, the pH was significantly reduced compared to before fermentation and the vitamin C content was significantly increased. (Mazlan et al., 2015) showed that the fermentation of bitter melon with *Lactobacillus plantarum* significantly increased the content of total phenols and total flavonoids, and the fermentation significantly changed the aroma of the juice, making it have a more ideal flavor.

**Table 2:** Conditions of the lactic acid bacteria fermentation process for fruit and vegetable juices.

Ingredients	Fermentation strain	Process condition	Final quality	References
Black wolfberry	*Lactobacillus bulgaricus*: *Bifidobacterium adolescens: Streptococcus thermophilus* = 1:1:1	T = 45℃、t = 15 h	The numbers of *Lactobacillus bulgaricus* and *Bifidobacterium* youth were (4.6 ∼ 5.6) × 1011 CFU·mL^−1^, (2.8 ∼ 3.8) × 1011 CFU·mL^−1^, pH = 3.95, and acidity were 20 mg· L^-1^ and 19 mg· L^-1^, respectively	Li et al., 2022
blueberry	*Lactobacillus casei: Lactobacillus plantarum* = 1:2	T = 36℃、t = 23 h	The lactic acid content was 8.33 g·kg^−1,^ and the total antioxidant capacity was 229.93U· mL^−1^	Li et al., 2021
Pumpkin or dragon fruit	*Lactobacillus casei: Lactobacillus plantarum* = 1:1	T = 37℃、t = 48 h	Viable bacteria number 2.30 × 109 CFU· mL^−1^, 3.79 × 109 CFU· mL^-1^pH = 3.36, 3.50, acidity: 133.38° T, 142.33° T	Zhao et al., 2015
Iron stick yam: Grape: tomato: carrot: apple = 5:1 :2:1:1	*Lactobacillus plantarum* CZ*: Lactobacillus plantarum* CA*: Lactobacillus plantarum* CB = 1 : 1 : 1, the inoculation amount was 6 %	T = 32 ℃、t = 8 h	Sweet and sour, delicate taste, uniform texture, no stratification, no bubbles, fermented flavor, orange-red, crystal color, apple, tomato, grape and light yam taste	Voaides et al., 2022
Banana pulp, sweet potato pulp, oligosaccharides	6 strains of probiotics were mixed in equal proportions	T = 38 ℃、t = 24 h	The number of viable bacteria is 9.58lg CFU· mL^−1^	Swain et al., 2014
Tomato to apple = 2 : 1	*Lactobacillus bulgaricus: Streptococcus thermophilus* = 1 : 1, inoculation volume 7 %	T = 43 ℃、t = 18 h	Light yellow transparent, uniform color, tomato and apple unique flavor, unique lactic acid flavor	Ricci et al., 2020
Fermented blueberries to tomatoes to carrots = 2:1:1	*Lactobacillus paracei* 2 × 107 CFU·mL^−1^ *Lactobacillus plantarum* 5 × 107 CFU·mL^−1^	T = 36℃, t = 6.45 h	pH = 3.8, the attractive dark purple color of blueberries	Cyrielle et al., 2020
Carrot, pumpkin, papaya, pineapple = 4:3 :1:2	The inoculated amount of direct injection *lactobacillus* was 0.2 %	T = 38℃、t = 22 h	Bright color, lubricated taste, unique flavor	Garcia et al., 2020

Composite strain fermentation can improve the quality of fruits and vegetables and produce more active ingredients through the synergistic action of multiple strains. (Bujna et al., 2017) inoculated mixed cultures of lactic acid bacteria and bifidobacterium in apricot juice, and the results showed that the number of viable bacteria in apricot juice fermented by mixed bacteria was higher after fermentation, and the concentration of acetic acid was higher about twice that of single bacteria fermentation, and the content of lactic acid was close to that of single bacteria fermentation. (Chen et al., 2023, Chen et al., 2023) used *saccharomyces cerevisiae* and *Lactobacillus plantarum* to ferment apple juice in stages, and the study showed that the contents of flavonoids, vitamin C and B6 increased after fermentation. After fermentation, ketones and acids were enriched, which significantly improved the flavor of apple juice. (Pontonio et al., 2019) fermented pomegranate juice with the combination of lactic acid bacteria, and the results showed that the combined fermentation of lactic acid bacteria significantly improved the quality of pomegranate juice, especially the combination of *Lactobacillus plantarum* and *Lactobacillus acidophilus* could significantly reduce the soluble solids pomegranate juice, increase the contents of total phenols and anthocyanins, and enhance the antioxidant effect of pomegranate juice. (Wang et al.,. 2022) studied the fermentation of pear juice by *Lactobacillus plantarum* 90, *Lactobacillus Swiss*-76 and *Lactobacillus casei* 37 under single and mixed fermentation conditions, and the results showed that the number of viable bacteria in pear juice by mixed fermentation was significantly higher than that by single fermentation. The content of vanillic acid and arbutin in fermented pear juice of *Lactobacillus plantarum* 90 and *Lactobacillus casei* 37 increased, and the DPPH ability to scavenge free radicals was stronger. (Kuerban et al., 2023; Mirmohammadi et al., 2021) fermented grape juice with *Lactobacillus plantarum* and *Lactobacillus brevis* at a ratio of 1:2, and the results showed that mixed bacterial fermentation could enrich phenolic compounds, enhance antioxidant capacity the grape juice has a characteristic aroma and a pleasant flavor. (Fonseca et al., 2021) explored the fermentation of bergamot juice by single and complex strains, and the results showed that the pH value, glucose and fructose consumption, lactic acid and formic acid generation, citric acid consumption and DPPH radical scavenging effect of fermented bergamot juice are affected by complex strains were significantly improved compared to fermentation of single strains. (Kuerban et al., 2023), It is shown that composite fermentation of fruit and vegetable products can not only enrich nutrients, and improve the flavor, however also promote functional activity. Fermentation of complex strains mainly involves asymbiosis or interaction between different strains and its metabolic mechanism is more complex than that of single strain fermentation, and the fermentation products are more abundant.

## Bioactive ingredients of fermented fruit and vegetable juice

The content of phenolic compounds, exopolysaccharides, B vitamins, and minerals in fruit and vegetable juices was significantly increased after fermentation (Chiou et al., 2017). These bioactive ingredients imparted many functional activities to fruit and vegetable juices, such as antioxidant activity, hypoglycemic activity and antihypertensive activity. The specific changes of the main bioactive components in fruit and vegetable juices after fermentation are presented in (Table 3).

**Table 3:** Fermentation-induced changes in bioactive compounds and functional activities of fruit and vegetable juices.

Ingredients	Fermentation strain	Active ingredients and functions	References
Black cherry	*Candida Lactobacillus, plantarum and Enterococcus faecium*	The organic acid produced after fermentation has an antibacterial effect on *Escherichia coli*, *Klebsiella pneumoniae* and *Pseudomonas aeruginosa*	Suriyaprom et al., 2022
mulberry	*Lactobacillus plantarum, Lactobacillus acidophilus and Lactobacillus paracei*	Eugenic acid, centaurin-3-O-rutin and quercetin significantly correlated with the ability to scavenge free radicals	Wang et al., 2015, Wang et al., 2015
Blueberry	*Lactobacillus plantarum*	After fermentation, the contents of phenols and anthocyanins in fruit juice were increased, and the clearance rate of 2,2-Diphenyl-1-picrylhydrazyl and superoxide anion radical was significantly increased, which could alleviate the oxidative damage of Caco-2 cells	Mauro et al., 2016
persimmon	*Flavobacterium and Lactobacillus plantarum*	The fermentation releases phenols, which greatly improve its ability to scavenge free radicals and inhibit carbohydrate hydrolase	Zhang et al., 2018
sugarcane	*Ganoderma lucidum*	The exopolysaccharide content was significantly increased and the ability to scavenge free radicals was evident	Lyu et al., 2022
Grapes	*Lactobacillus plantarum*	γ-aminobutyric acid produced by fermentation has some antihypertensive effect	Diez-Gutiérrez et al., 2022
pomegranate	*Lactobacillus plantarum*	The angiotensin converting enzyme inhibitory activity of pomegranate juice was significantly increased by fermentation, which was related to the release of phenolic substances	Chen et al., 2023, Chen et al., 2023
Cashew apple	*Lactobacillus acidophilus and Lactobacillus casei,* etc	The fermentation of *Lactobacillus acidophilus* and *Lactobacillus casei* could increase the content of B vitamins by 19.25 % and 23.11 %, respectively	Kaprasob et al., 2018
carrot	*Lactobacillus plantarum*	The fermentation creates short-chain fatty acids that promote the secretion of Glucagon-like peptide 1and thus achieve a blood-sugar-lowering effect	Li et al., 2014

### Analyzing phenolic compounds in commercial fruit and vegetable juices.

**Fig. 3:**
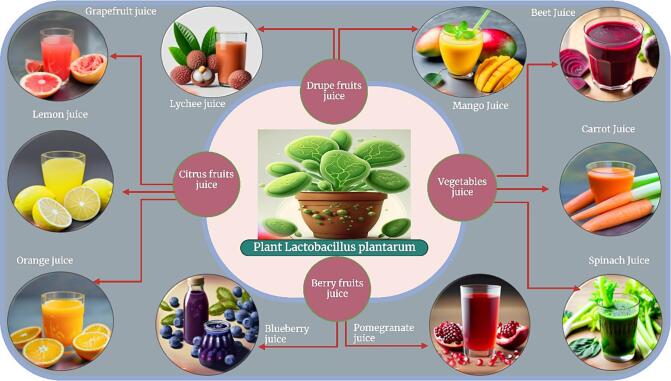
Types of *Lactobacillus plantarum* fermentation of fruit and vegetable juices.

Phenolic compounds are natural antioxidants found in fruits and vegetables that provide protection against diseases related to oxidative stress. In the natural fermentation process of bergamot melon, the content of flavonoid substances such as luteolin, apexin, geranylline and isorhamnetin increased significantly. A correlation analysis revealed that eight phenolic compounds were highly correlated with the increase in antioxidant activity (Martínez et al., 2021). The dynamic changes of phenolic compounds during fermentation were studied in various foods and showed changes in composition and antioxidant properties. Fermentation can lead to the release of bound phenolics and the biotransformation of phenolic compounds by microorganisms, which ultimately influences the bioactivity and bioavailability of the resulting fermented foods (Melini and Melini, 2021, Yang et al., 2023).. However, the fruit juice is rich in substrates necessary for the fermentation of *Lactobacillus plantarum* and the antioxidant substances it contains can promote the establishment of an anaerobic environment and the pH of the juice is also suitable for the survival and proliferation of *Lactobacillus plantarum* under suitable fermentation conditions provided such that the growth of *Lactobacillus plantarum* follows the microbial growth curve in most fruit juices (Fig. 3). Ferulic esterase produced by *Lactobacillus plantarum* can catalyze the release of ferulic acid from conjugated phenolic acid, and tannase can metabolize catechin and gallic acid into protocatechin acid. The release and transformation of phenolic substances are the reasons for the improvement in antioxidant activity of kiwifruit juice after fermentation. (Zhou et al., 2020) observed that the total phenolic content of mulberry juice was significantly increased after fermentation by lactic acid bacteria, which was attributed to the hydrolysis of complex phenols into small phenol molecules by lactic acid bacteria hydrolase. The difference in total phenolic content of different strains was related to the diversity of hydrolase produced by the strains, leading to the difference in hydrolytic ability. (Tlais et al., 2023) explored the influence of fermentation of lactic acid bacteria on the change of phenolic content in sauerkraut and found that lactic acid bacteria metabolized phenolic acid through phenolic acid reductase or decarboxylase, converting caffeic acid into dihydrocaffeic acid and 4-ethyl catechol, ferulic acid into dihydroferulic acid, and caprylic acid into 4-vinylacetopol. 3-hydroxybenzoic acid is converted to catechol, and the derivative exhibits better antioxidant activity than the precursor. In short, the increase in the content of phenolic substances in fruits and vegetables after fermentation is related to the degradation of plant cell wall resulting in the release of phenolic substances; on the other hand, it is related to the hydrolysis of macromolecular phenolic substances and the transformation of phenolic substances by various enzymes into microorganisms.

### Exopolysaccharide production in fermented fruits and vegetables“

Extracellular polysaccharide is a secondary metabolite secreted outside the cell wall during the growth process of microorganisms. It not only can improve the rheological properties of fermented fruit and vegetable juice, but also has various biological activities such as anti-oxidant, anti-tumor and immune regulation (Swain et al., 2014). At present, the synthesis mechanism of exopolysaccharides mainly includes four steps: the entry of monosaccharide molecules into the cytoplasm, the synthesis of glucose-1-phosphate, the synthesis of exopolysaccharide repeating units, and the polymerization of exopolysaccharides (Nguyen et al., 2020). Many studies have confirmed that fruits and vegetables can produce exopolysaccharides after fermentation. (Lyu et al., 2022) used sugarcane juice as raw material and fermented sugarcane juice with *ganoderma lucidum* at 28 °C and found that after fermentation, the content of extracellular polysaccharide and protein were significantly increased, and free amino acids were significantly enriched. The antioxidant activity test showed that exopolysaccharide has the ability to scavenge hydroxyl radicals, superoxide anion radical and DPPH (Diphenyl-1-picrylhydrazyl) radical. (Chen et al., 2019) evaluated the effect of co-fermentation of *Lactobacillus rhamnosus and Glucosus xylosus* on the quality of litchi longan juice under high hydrostatic pressure, and the results showed that the yield of exopolysaccharides after fermentation was higher than that of unfermented juice.

### Vitamins

Fruits and vegetables are rich in vitamin C, however low in vitamins B, which, as a coenzyme, plays a key role in various reactions in the body's metabolism. It has been reported that fermentation can boost the production of B vitamins. (Kaprasob et al., 2018) used five kinds of lactic acid bacteria to ferment cashew apple juice and explored the effect of fermentation bacteria on the production of B vitamins. They found that fermentation of *Lactobacillus acidophilus* and *Lactobacillus casei* increased B vitamins levels by 19.25 % and 23.11 %, respectively. (Presti et al., 2015) examined new strains of *Lactobacillus* and *Bifidobacterium* which can also produce B vitamins. (Russo et al., 2014) used a single or compound fermentation of pineapple pulp with *Lactobacillus casei*, *Lactobacillus plantarum* and *Leuconostoc intestinalis* and found that vitamin C content increased in the single fermentation group of *Leuconostoc intestinalis* compared to that before fermentation, while it increased the other groups decreased to varying degrees. (Szutowska et al., 2021) carried out natural fermentation of kale and found that vitamin C content decreased significantly after fermentation. The reason for the decrease in vitamin C content caused by fermentation may be related to its own instability and easy oxidative decomposition or related to the metabolic utilization of lactic acid bacteria in the fermentation process.

### Other substances

The low bioavailability of minerals in fruit and vegetable juice is mainly due to the fact that minerals can easily to be complexed with other substances, and the complex can be degraded through to release minerals. (Quan et al., 2022, Sevindik et al., 2022) found that fermentation by lactic acid bacteria significantly increased the total mineral content of viburnum sap, particularly the elements potassium, magnesium, copper, iron and zinc. The increase in mineral content may be due to the decomposition of mineral- polyphenols in viburnum juice due to fermentation, releasing small molecules of phenols and minerals. In addition, it has been reported that fermentation can convert proteins in fruits and vegetables into bioactive peptides. As we all know, bioactive peptides are functional factors with great potential for commercial application, and have many beneficial effects such as lowering blood pressure, anti-oxidation and immune regulation. (Aredes et al., 2011) found that *Oenococcus oeni* has proteolytic activity and can release a variety of beneficial bioactive peptides from grapes, thereby providing antioxidant and antihypertensive effects.

## Functional activities of fermented fruit and vegetable juice

**Fig. 4:**
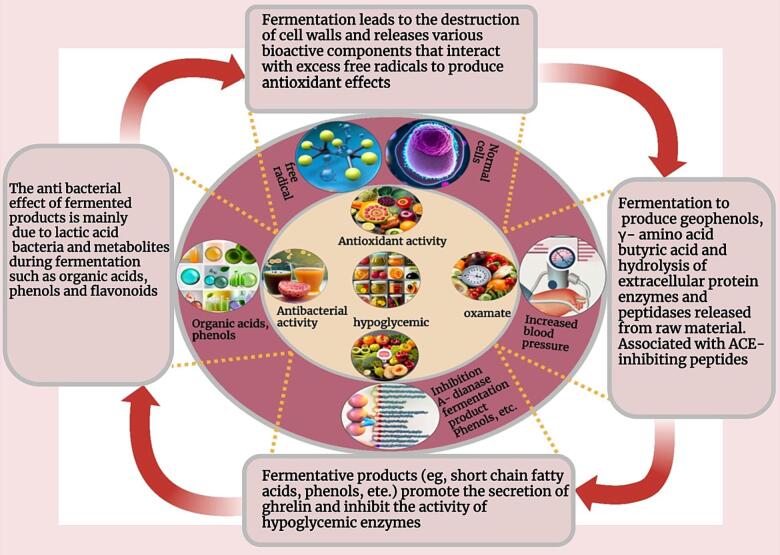
The beneficial aspects of lactic acid bacteria fermented fruit and vegetable juices.

After fermentation, fresh fruit and vegetable juice has a variety of functional activities that are beneficial to human health, such as: antioxidant activity, hypoglycemia, blood pressure and antibacterial activity (Fig. 4) which are closely related to formation of linked are fermentation products as a phenolic compounds, exopolyaccharides and vitamins.

### Nutritional health benefit of vegetable/fruit juice

Fruit and vegetable juices fermented with probiotics have nutritional and health functions. (Yang et al., 2022, Yang et al., 2022) fermented silver almond juice with *Lactobacillus acidophilus*, *Lactobacillus plantarum* and *Lactobacillus paracei*. The number of lactic acid bacteria in silver almond juice reached over 8.0 log CFU/ml, the content of terpenolactone increased 1.6-fold, the total amount of phenolic substances increased by about 9 %, and the ability of ABTS to scavenge free radicals increased by 35.3 %∼39.8 % can effectively inhibit the growth of pathogenic bacteria such as *Escherichia coli*. (Shaban et al., 2021) found that fermented papaya juice had a certain protective effect on CTC-induced liver damage, reflecting the activities of low pyrulol transaminase and glutamic-oxaloacetic transaminase, as well as the mRNA levels of TGF-lung, TLR4 and IL-1 in liver tissue could reduce and improve the health of mouse liver tissue. (Ranadheera et al., 2017, Sharma and Mishra, 2013) It has been found that fermented fruit juice can retain the maximum nutrients of the fruit and the fermentation process can even produce more nutrients, improving the intestinal environment and immunity. In addition, the effects of fermented noni juice were studied in diabetic mice and showed that it can lower blood sugar and protect the liver. This is consistent with the general benefits of fermenting fruits and vegetables, which include improved preservation, a unique flavor profile, increased nutritional value, and support for immune responses and gut microbial diversity. Probiotic fermentation technology offers a promising approach to increase the health benefits of fruit and vegetable products and make them a valuable addition to a healthy diet.

**Fig. 5:**
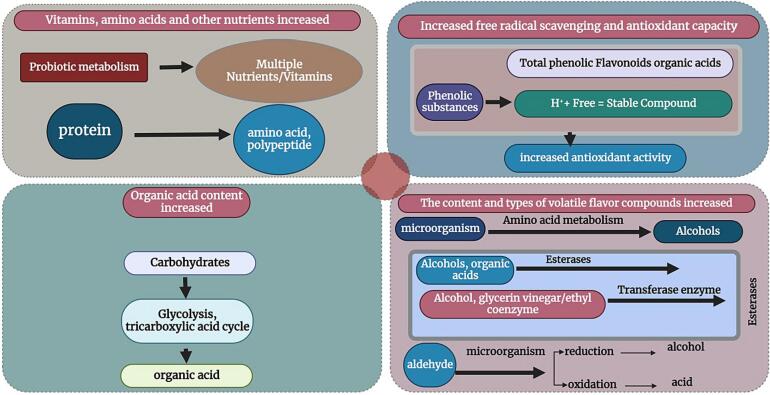
Influence of fermentation on the nutritional quality of fruit and vegetable juice.

The microbial metabolism in the fermentation process changes the content and composition of the enzyme system of the juice and thus also the quality of the juice, such as flavor, elimination of free radicals, antioxidants, etc (Sharma et al., 2020; Yang et al., 2020). The most important substance changes that fermentation brings about in juice are shown in (Fig. 5) (Leeuwendaal et al., 2022). Some studies have found that after fermentation by lactic acid bacteria, the contents of total phenols, flavonoid vitamins, minerals and exopolysaccharides in fruit juice increase.(Maia et al.,2023; De et al.,2023) studied the substance changes of 5 different fruit and vegetable juices before and after fermentation, and found that the contents and types of volatile flavor substances, aromatic amino acids and benzene-derived flavor substances increased, while the aldehydes and free amino acids decreased, indicating that the free amino acids may decompose into some small molecules of volatile flavor substances during fermentation. (Yang et al., 2018) found that mango juice fermented by lactic acid bacteria increased total phenol content, antioxidant activity and juice quality. (Li et al., 2018) fermented star fruit juice with *Lactobacillus Swiss*, *Lactobacillus paracasei* and *Lactobacillus rhamnosus* respectively, and found that the contents of aldehydes and vinegar in star fruit juice decreased significantly, while the contents of ketones, alcohols and fatty acids increased to a certain extent, which improved the flavor of star fruit juice. (Yang et al., 2018, Pontonio et al., 2019) found that the antioxidant activity of mixed pulp juice was significantly increased after fermentation with lactic acid bacteria, and 34 new volatile compounds were added.

### Effects of probiotic fruit and vegetable juices on the immunoregulatory function

Fermented fruit and vegetable juice contains calcium, zinc, iron, copper and other trace elements, which can promote human bone development, promote gastrointestinal peristalsis and regulate immune function. It has been reported that during the fermentation process, the hyaluronic acid inhibition rate of pomegranate juice in the fermentation group was always higher than that in the non-fermented group, and its anti-allergic ability was increased 2.68 times at the end of fermentation (Basiri et al., 2013). Fermented carrot juice and pear juice can enhance the proliferation ability of mouse spleen lymphocytes, increase the expression levels of CD3, CD4 and CD8, and promote the action of serum IFN- and the production of IL-6, thus improving the immunity of mice (Muhialdin et al., 2021, Dębińska and Sozańska, 2022).

### Prevention of cardiovascular diseases

Polyphenols in fermented fruit and vegetable juices have bidirectional regulation of lipid metabolism, and oligosaccharides or dietary fibers can be used as probiotics to reduce blood lipids by regulating the growth of microorganisms in the colon. (Kim et al., 2012) found that fermented fruit and vegetable juice could inhibit angiotensin-converting enzyme activity thereby reducing the probability of cardiovascular disease. It has also been reported that lactic acid bacteria and its related products can lower cholesterol levels in media and serum, and reduce the risk of cardiovascular diseases (Lee et al., 2009).

### Antioxidant properties of fermented fruit and vegetable juices

**Fig. 6:**
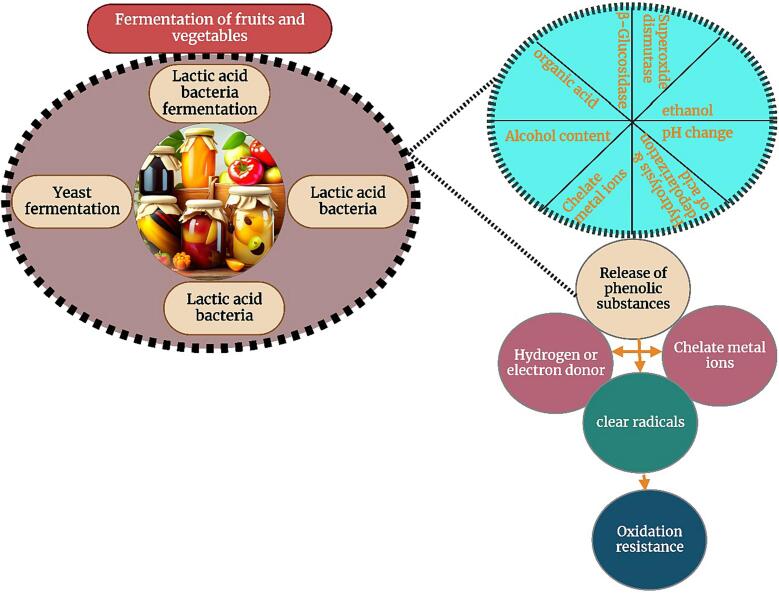
Antioxidant properties of fermented fruit and vegetable beverage.

In the process of metabolism, human body produces a large number of free radicals and reactive oxygen species, which are closely related to many diseases. Due to the safety concerns associated with synthetic antioxidants, the development of fermented foods with the ability to scavenge free radicals is important to improve human health. The antioxidant mechanism of fermented fruit and vegetable juice is shown in (Fig. 6). The fermentation of fruit and vegetable juices leads to the destruction of plant cell wall structure and releases various bioactive ingredients, which interact with excess free radicals to achieve the antioxidant effect. Phenolic compounds, carotenoids and anthocyanins are the main antioxidant substances in fruits and vegetables, which can play an antioxidant role as reducing agents, hydrogen donors and singlet oxygen quenching agents. (Li et al., 2021, Yu et al., 2023) used *Lactobacillus plantarum* to ferment blueberry juice, and the results showed that fermentation significantly increased the clearance rates of DPPH and superoxide anion free radicals, and had an alleviating effect on the oxidative damage of Caco-2 cells. (Wang et al., 2023, Aleman et al., 2023) studied the effects of fermentation of six commercial lactic acid bacteria on the active phytochemicals, antioxidant activity and flavor in wolfberry juice, and the results showed that the antioxidant activity based on DPPH, ABTS and FRAP methods according to LAB fermentation has been significantly improved. The improvement of antioxidant activity of wolfberry juice is the synergistic effect of various antioxidants in wolfberry juice, such as phenolic acids, flavonoids and vitamins. Correlation analysis showed that rhizomycin and p-coumaric acid were positively correlated with DPPH and ABTS free radical scavenging activities. The mulberry juice was fermented by *Lactobacillus plantarum*, *Lactobacillus acidophilus* or *Lactobacillus paracei* at 37 °C for 36 h. The main phenolic acids in *Lactobacillus* fermented mulberry juice were syringic acid, centaurin-3-O-rutin and quercetin, which had a significant correlation with free radical scavenging ability (Kwaw et al., 2018).

### Anticancer potential of fermented fruit and vegetable juices

When there are too many reactive oxygen species in the human body, carcinogens are activated and the intracellular antioxidant defense system is inhibited (Ruiz et al., 2019). During the fermentation of fruit and vegetable juices, probiotics produce other antioxidant substances such as polyphenols, which can effectively remove free radicals in the body. (De et al., 2023) selected different lactic acid bacteria to inoculate longan juice. By measuring total phenol, bound phenol, free phenol and flavonoid levels before and after fermentation, they found that different lactic acid bacteria can increase total phenol and flavonoid contents, as well as antioxidant capacity, to varying degrees. Some studies have reported that exopolycans produced by lactic acid bacteria fermentation can inhibit tumors and improve the body immunity (Sørensen et al., 2022). Improving the safety of fruit and vegetable juices; extend shelf-life. In addition to the biological protective effects, mentioned above, probiotics can also improve the safety of fruit and vegetable juices. In the fermentation of fruit and vegetable juices, probiotics, as the dominant group of bacteria, can establish a competitive inhibition relationship with other microorganisms and inhibit the proliferation of other microorganisms. For example, many fruits and vegetables contain a certain amount of nitrate, during the fermentation process, some hybrid bacteria can convert nitrate into nitrite, and also produce nitrite amine, which is harmful to the human body. However, studies have found that lactic acid bacteria can reduce nitrite levels in fermented fruit and vegetable juice (Yang et al., 2018) used carrot and papaya as raw materials and selected plant-based DMDL 9010 from *Lactobacillus* for the fermentation of mixed fruit and vegetable juice. The optimal process conditions were adopted, that is, the juice content of fruits and vegetables was 65 % (v/v) the mass ratio of carrot to papaya was 1:3, the sucrose content was 9 % (v/v), and the inoculation rate was 7 % (v/v). The shelf life of the developed viable and sterile fruit and vegetable juice can be thirty days and three months respectively, which greatly extends the shelf life of fruit and vegetable juice.

### Uncovering the hypoglycemic potential of fermented fruit and vegetable juices

Currently, hypoglycemic drugs in clinical use have great side effects. Researchers began looking for blood-sugar-lowering drugs without the toxic side effects of natural functional foods. Many studies have proved that fermented fruit and vegetable juices have a blood-sugar-lowering effect. This was related to the fact that post-fermentation products (such as short-chain fatty acids, phenols, etc.) promoted the secretion of enter in hormones and inhibited carbohydrate hydrolase activity. (Nayak et al., 2010) found that fermented noni juice had hypoglycemic and hepatoprotective effects in diabetic rats, and speculated that it might be a substitute for glibenuride, an excellent hypoglycemic drug. (Wu et al., 2023, Li et al., 2014) fermented carrot juice with *Lactobacillus plantarum*, and the results showed that the strain promoted the production of short-chain fatty acids in the fermented juice, resulting in a higher proportion of short-chain fatty acid content. Chain fatty in the colon of rats with type II diabetes. Short-chain fatty acids can promote the secretion of glucagon-like peptide-1 (GLP-1), an intestinal secretin hormone, and its main function is to stimulate the release of glucose-dependent insulin and promote glucose homeostasis, thus achieving the effect of anti-diabetes. α-glucosidase and α-amylase are two important carbohydrate hydrolases, and inhibition of the activity of these two enzymes will reduce the production of free glucose, thereby regulating human blood sugar. (Wu et al., 2023) screened lactic acid bacteria with hypoglycemic function and added them into apple juice for fermentation. The results showed that the inhibition ability of fermented apple juice on α-glucosidase was significantly higher than that of unfermented apple juice, which may be related to exopolysaccharides produced by lactic acid bacteria, which can interact with enzymes through electrostatic attraction or hydrogen bonding. This changes the structure of the enzyme, which leads to reduced activity. (Plessas, 2022) fermented blueberry juice with *Lactobacillus plantarum*, and found that the inhibitory ability of blueberry juice to α-glucosidase and α-amylase was enhanced after fermentation. This result was related to the increased content of phenolic substances by the fermentation of *Lactobacillus plantarum*, since polyphenols can effectively inhibit the enzyme activities of α-glucosidase and α-amylase. Studies have shown that polyphenols form stable complexes with enzymes through hydrogen bonding and hydrophobic interaction, thus reducing their catalytic activity. (Zhang et al., 2018, Wang et al., 2022) found that the inhibitory activity of persimmon fermented by *flavobacterium* and *Lactobacillus plantarum* on α-glucosidase was 2.06 ∼ 4.73 times that of the non-fermented group and the inhibitory effect on the α-glucosidase activity was highest at a fermentation time of 48–72 h.

### Exploring the antibacterial effects of fermented fruit and vegetable juice

Studies have shown that fermented products have an antibacterial effect on both gram-positive and gram-negative bacteria. (Gumienna et al., 2016) found that fermentation of pomegranate juice by *Lactobacillus plantarum* could improve its antibacterial activity compared with unfermented fruit juice. The combination of fermented cranberry juice and antibiotics has an antibacterial synergistic effect. Metabolites of lactic acid bacteria and phenolic compounds in cranberry juice help to better inhibit the growth of pathogenic bacteria (Loanna et al., 2019, Vanitha et al., 2023, Divyashree et al., 2023) studied the inhibitory activity of Candida *Lactobacillus plantarum MYSAS1* and *Enterococcus faecium* MYSAS4 isolated from fermented black cherry fruits against *Salmonella enterica*. The results showed that these two kinds of *lactobacillus* had inhibitory effects on *Escherichia coli*, *Klebsiella pneumoniae*, *Pseudomonas aeruginosa*, *Staphylococcus aureus* and *Salmonella paratyphi*, especially the inhibition rate of *Lactobacillus plantarum* could reach more than 80 %. (Ayed et al., 2017) found that fermented red grape juice had antibacterial activity against seven species of pathogenic bacteria (such as *Staphylococcus aureus*, *Staphylococcus epidermidis* and *Escherichia coli*), which is mainly due to the increase of acetic acid content in the fermentation process. The antibacterial potential of fermented fruit and vegetable juice is mainly due to the metabolites with antibacterial activity produced during the fermentation of lactic acid bacteria, such as organic acids, fatty acids, hydrogen peroxide or diacetyl, and some antibacterial proteins, such as bacterin peptidoglycan hydrolase. The bacteriostatic mechanism of organic acids mainly involves the release of protons after the undissociated acids enter the cells, thus reducing the pH value of the cytoplasm. In addition, undissociated acids can also change the permeability of cell membranes, resulting in the death of pathogenic bacteria (Szutowska et al., 2020). Studies have shown that flavonoids can disrupt the interaction between acyl homoserine lactones and their receptors, and also inhibit the surface adhesion of gram-positive bacteria (Shamsudin et al., 2022).

### Role of fermentation in enhancing hypotensive activity of fruit and vegetable juice

The survey shows that the prevalence rate of hypertension among the elderly over 65 years old in China has reached 64.23 % (Song et al., 2019), and a large number of studies have shown that fermented fruit and vegetable juice has a potential effect on health have lower blood pressure. After fermentation by *Lactobacillus plantarum*, persimmon juice significantly enhanced the activation of alcohol dehydrogenase and acetaldehyde dehydrogenase as well as the inhibitory activity of angiotensin-converting enzyme, showing potent alcohol inhibitory and antihypertensive effects (Wang et al., 2016; Kurt et al., 2006) found that fermented berries have antioxidant potential and an ability to relax vessels. Studies have also shown that gamma-aminobutyric acid may be linked to preventing high blood pressure, however it is rarely found in natural fruits and vegetables, and through fermentation, l-glutamate can be converted to gamma-aminobutyric acid. (Di et al., 2010) found that *Lactobacillus plantarum* synthesized 4.83 mmol/L gamma-aminobutyric acid during fermentation of grape juice at 30 °C for 72 h. (Lin et al., 2016) studied the inhibitory activity of fermented pomegranate juice on the conversation of angiotensin converting enzyme (ACE),and the results showed that fermentation of *Lactobacillus plantarum* f significantly improved the ACE inhibitory activity of pomegranate juice. Further studies showed that the ACE inhibitory activity of pomegranate juice is mainly related to phenols, and the ACE inhibitory activity of epicatechin and chlorogenic acid in pomegranate juice was more than 30 %. Phenolic compounds passivated the catalytic activity of ACE by interacting with amino acids or zinc ions at the active site (Upadrasta et al., 2016). By hydrolyzing their extracellular protease and peptidase, lactic acid bacteria can also release ACE inhibiting peptides in the raw material matrix,and thus have an antihypertensive effect. The bacterial components such as exopolysaccharides of lactic acid bacteria can also play a role in hypotensive activity.

### Other functional activities

(Xu et al., 2023) selected four kinds of *Lactobacillus acidophilus* and *Lactobacillus rhamnosus* to fermentation of *Lentinus edodes* and found that the fermentation products could inhibit the activities of α-amylase, α-glucosidase and pancreatic lipase, as well as the metabolites such as nitrite and prostaglandin lip-induced inflammatory reactions. Therefore, it has the potential to be developed as a functional probiotic supplement or food. The antithrombotic activity of gallnut fermentation liquid was tested in vitro. The results showed that the antithrombotic mechanism of gallnut fermentation liquid was accelerating fibrinolysis and blood clot degradation and prolongation of prothrombin time. In vivo experiments have also shown that thrombus formation has been alleviated and paralysis or death from thromboembolism has been avoided to a certain extent (Balakrishnan et al., 2020) studied whether daily intake of fermented citrus juice with hot bactericidal *Lactobacillus vegetarium* could alleviate the symptoms of atopic dermatitis, and the results showed that fermented citrus juice can relieve a variety of allergic diseases.

## The improvement effect of probiotics on fruit and vegetable juice

### Probiotication of intestinal health

During the fermentation of fruit and vegetable juices, Probiotics produce a large amount of organic acids which not only lower the pH value in the gut, leading to a local acidic environment, but also reduce the REDOX potential. These effects work together to maintain the microecological balance in the gut. (Valero-Cases et al., 2017) found through research that probiotic fermented fruit and vegetable juices could help improve the probiotic adhesion ability of drinkers in the gut. (Behera et al., 2018) showed that fruit and vegetable juice fermented by *Lactobacillus plantarum* can significantly improve the microecological milieu in the gastrointestinal tract and increase intestinal motility.

### Enhance the flavor volatile compounds of fermented vegetable and fruit substrates

Microorganisms can use precursor such as free amino acids, fatty acids and sugars to synthesize flavor compounds in the fermentation process, resulting in fruity, ester and floral flavors (Table 4.). Flavor compounds are one of the most important indicators for consumers to evaluate fruit juices, and are closely related to the sensory quality of fermented fruit and vegetable products. (Guiné et al., 2021) used *Kluyveromyces lactatus* and *Lactobacillus plantarum* to ferment apple juice concentrate and found that the amino acid content of Kluyveromyces lactatus decreased significantly after fermentation, which was due to the strain using amino acids to synthesize higher alcohols, thus giving fermented apple juice a pleasant aroma. In addition, valine, methionine, leucine and other bitter amino acids were metabolized by the yeast fermentation, which had a positive effect on improving the flavor of fermented apple juice. A low concentration of aldehydes can produce an acceptable flavor, while a high concentration produces an odor. (Kim et al., 2023) found that the fermentation of bitter melon juice by *Lactobacillus plantarum* can reduce the content of odors aldehyde, while increasing alcohol and acid, so that it has a more ideal flavor. The aldehydes content in wolfberry juice decreased from 1841.22 mg/L to 332.83 mg/L of the *Lactobacillus* plant, eliminating the bad taste. Esters play an important role in improving the flavor of fruit and vegetable juices, and are generally formed from the esterification of acids and alcohols produced after fermentation. The fermentation of lycium berry juice by *Lactobacillus* Swiss resulted in the accumulation of fruity esters, especially the highest content of ethyl acetate, which was the result of the synthesis of acetyl-CoA and acetyltransferase (Wu et al., 2022) in the fermented sea buckthorn juice with four species of lactic acid bacteria, and the results showed that after fermentation by lactic acid bacteria, the acidity of sea buckthorn juice was significantly improved, while bitterness and astringency were significantly reduced. After fermentation by *Lactobacillus plantarum*, the volatile content in Seabuck Buckans juice was the highest, especially a large number of esters, such as ethyl caproate, isoamyl isovalerate, ethyl capryl, ethyl benzoate, ethyl butyrate, etc., and the sensory evaluation of *Lactobacillus plantarum* group was also the highest. (Perricone et al., 2015) fermented grape juice by inoculating the mixture of *Lactobacillus plantarum* and *Lactobacillus brevis*. The study showed that the contents of monoterpene and laurene increased after fermentation. They are important aromatic compounds with floral and fruit aroma, making the flavor of juice more popular among consumers. The increased terpene content may be due to hydrolysis of conjugated precursors in grape juice by β-glycosidase.

**Table 4:** Flavour volatiles in fermented fruit and vegetable juice.

Ingredients	Fermentation strain	volatile substance	fragrance	References
Hawthorn	*Lactobacillus casei, Lactobacillus paracasei, Bifidobacterium, etc*	With the increase in species and total amount of volatile components, 17 new alcohols and new esters were formed and the aldehyde content decreased	Clove, rose, wood, fruit, ester, etc	Wang et al., 2015, Wang et al., 2015
Chinese wolfberry	*Lactobacillus plantarum, Lactobacillus paracei, Lactobacillus acidophilus, etc*	Aldehyde content decreased; The contents of acids, esters and alcohols increased, such as 2-methylbutyric acid, heptanoic acid, 1-octanol, 1-hexanol, ethyl acetate, ethyl pelanoate, etc	Fruit, sweet, bread, grass, honey, pineapple, grape, etc	Wang et al., 2023
Pear	*Lactobacillus plantarum, Lactobacillus Swiss, Lactobacillus casei*	The contents of alcohols such as linalool and (E) −2-hexene-1-ol and esters such as ethyl acetate and ethyl butyrate were significantly increased	Floral, fruity, apple, rose, etc	Wang et al., 2021
Grapes	*Lactobacillus plantarum and Lactobacillus brevis*	total volatiles including alcohols, esters, acids and terpenes increased by 67.00 %	Floral and fruity aromas	Mirmohammadi et al., 2021
Cashew apple	*Lactobacillus acidophilus, Lactobacillus casei, Lactobacillus plantarum*	The contents of alcohols and esters increased after fermentation, including 2, 6-dimethyl-4-heptanol, phenylethanol, phenylpropanol, ethyl 3-methylbutyrate, ethyl caproate, etc	Cream, whiskey, sweet, sour, cheese, fruit, fat, etc	Liu et al., 2021
Bitter melon	*Lactobacillus plantarum*	The contents of 1-hexanol, benzaldehyde, hexadecanoic acid and caprylic acid were significantly increased after fermentation.	Floral, almond, caramel, sweet, cheesy, herbal, etc	Mahwish et al., 2021
Apple	*Lactobacillus Swiss, Lactobacillus acidophilus, Lactobacillus casei, etc*	7 new alcohols were produced, including 2-methyl-1-propyl alcohol, isobutyl carbinol, benzyl alcohol, citronellol, geraniol, etc. Six new esters, including ethyl acetate, ethyl propionate, amyl acetate, etc	Rose, citrus, fruity, sweet, etc	Liu et al., 2021
Sea buckthorn	*Lactobacillus plantarum*	The content of esters, such as ethyl caproate, isoamyl isovalerate, octyl, was significantly increased, Ethyl acid, ethyl benzoate, ethyl butyrate, etc	Fruit, apple, pineapple, banana, sweet, etc	Li et al., 2022

### Improve the nutritional value of fruit and vegetable juice

The oligosaccharides formed in the fermentation process of fruit and vegetable juices can act as bifidus factor to balance the human intestinal flora and play a nutritional role in the human body. Studies have shown that after fermentation of the mixture of citrus juice, apple juice, pear juice and carrot juice by Bifidobacterium, the content of vitamin C, beta-carotene and folate increased by2-, 1.5-, and 2.5-fold, respectively (Rodrigues et al., 2022). Polysaccharides in fruit and vegetable juices are hydrolyzed to produce useful components in the fermentation process, such as oligosaccharides which can be used as bifidus factors for human nutritional and health functions (Fonteles et al., 2018). It has been reported that after yeast fermentation, the nutritional value of fruit and vegetable juices improves and is easier to absorb, mainly during yeast fermentation, amino acids, B vitamins and other active compounds (such as glutathione and ergosterol) can be formed from fruit and vegetable juices, which improves the nutritional value of the product (Pang et al., 2022).

### Supplement probiotics

As a non-dairy probiotic beverage, probiotic fermented fruit and vegetable juice is of great benefit to some special consumers, especially strict vegetarians, lactose intolerant and hypercholesterolemia, and thus occupies a large market share. Probiotic drinks are the main way for most consumers to supplement with probiotics. Studies have shown that the number of viable bacteria of probiotic drinks should reach more than 106 ∼ 107 CFU/mL for better health effect (Maia et al., 2023, Mojikon et al., 2022), and it also needs to maintain good activity during the shelf life. However, it is challenging to ensure the above requirements. In recent years, some researchers have found that adding an appropriate amount of prebiotics can have a symbiotic effect with probiotics, thus maintaining a high number of viable bacteria in probiotic beverages and improving their sensory quality, physical and chemical properties (Yang et al., 2022, Yang et al., 2022).

### Influence of lactic acid fermentation on the shelf life of fruit and vegetable juices

Lactic acid fermentation can extend the shelf life of food mainly due to the synthesis of a variety of metabolites, such as organic acids, carbon dioxide, ethanol, diacetyl and hydrogen peroxide, which have an inhibitory effect on a variety of harmful bacteria. (Muhialdin et al., 2020) found that it was fermented by *Lactobacillus plantarum*. Dragon juice greatly improved the antibacterial activity against *Escherichia coli, Salmonella typhimurium*, *Pseudomonas aeruginosa* and *Staphylococcus aureus*, and the shelf life of dragon juice was longer than that of unfermented dragon juice for three months. (Alegre et al., 2011) found that *Lactobacillus rhamnosus* significantly inhibited salmonella and listeria during apple storage, thus extending the shelf life of apples. Another study found that adding fermented cantaloupe juice to fresh cantaloupe juice can extend the shelf life for 6 months under 8 °C storage conditions, and fermented cantaloupe juice has strong antibacterial activity against *Escherichia coli, Salmonella typhimurium, Aspergillus flavus* and *Penicillium* (Liu et al., 2023).

### Role of lactic acid fermentation in reducing nitrate content in fruit and vegetable juices

Traditional fermentation methods, such as fermenting vegetables with high salt content, generate a large amount of nitrite in the fermentation process, which affects the safety of fermented products. Studies have shown that *Lactobacillus*, *Leuconostoc* and *Micrococcus* have the function of degrading nitrite, and the main degradation pathways are enzymatic hydrolysis and acidolysis. Studies have also shown that lactic acid bacteria can break down nitrite in other ways, such as inhibiting the growth of pathogenic bacteria such as *Escherichia coli*, thereby reducing nitrite production and accumulation. (Xia et al., 2022) found that fermentation of lactic acid bacteria resulted in increased acidity and nitrite was easily degraded in a low pH environment, reducing its content. (Alan et al., 2019) prepared cabbage cucumbers by inoculating *Lactobacillus plantarum* and *Lactobacillus rhamnosus*. The study showed that the best flavor was obtained at a seeding rate of 3 %, a fermentation temperature of 30 °C, a salt addition rate of 4 % and the nitrite level was well below the product standard.

## Conclusion and prospect

The development of fermented fruit and vegetable juice can better solve the problem of wasting fruit and vegetable resources, and improve the value-added and utilization rate of fruit and vegetable processing by-products. Currently, three fermentation methods are mainly used to ferment fruit and vegetable juices: natural fermentation, inoculation of internal strains isolated from fruits and vegetables, and fermentation of commercial strains. After fermentation, fruit and vegetable juice produces rich bioactive substances with various functional activities such as antioxidant, hypoglycemic, antihypertensive and antibacterial effects, etc., and its quality has been greatly improved, such as: B. Improving original taste, extending shelf life, reducing nitrite levels. However, there are also significant problems in the fermented fruit and vegetable juice industry, such as: B. the incomplete understanding of the mechanism of functional activity and the lack of relevant clinical studies. Using molecular biology, genomics and metabolomics, the specific regulatory mechanisms of the active ingredients in fermented fruit and vegetable juices and their functional properties can be further elucidated. In addition, the fermentation technology is not perfect, the safety of the product needs further verification, and relevant industry standards are lacking. As people pay more and more attention to their own health problems, fermented fruit and vegetable juice will be an important part of the functional food market in the future, with a long-term trend towards sustainable development. Therefore, future research should continue to pay attention to the above technical shortcomings. At the same time, more fermented fruit and vegetable juice products with good flavor and health benefits have been developed to meet market demand.

Ethical approval

Not applicable.

Consent to participate.

All authors are agreed to contribute to this study.

Consent to publish.

Not applicable.

## Funding

No funding was obtained for this study.

Availability of data and materials.

Not Applicable.

There is no Declaration of Interest Statement.

## CRediT authorship contribution statement

**Shah Saud:** Conceptualization, Writing – original draft, Writing – review & editing. **Tang Xiaojuan:** Conceptualization. **Shah Fahad:** Conceptualization, Writing – original draft, Writing – review & editing.

## Declaration of competing interest

The authors declare that they have no known competing financial interests or personal relationships that could have appeared to influence the work reported in this paper.

## Data Availability

No data was used for the research described in the article.
